# 

**Published:** 1904-05-15

**Authors:** 


### CONTENTS.

Inlay Cavity Preparation, - *By F. E. Roach. D.D.S.* 217

Alloys of Commerce and their Metal Formulæ, *By N. K. Garhart, Ph.G.* 225

Seamless Crowns, - - *By M. L. Ward, D.D.S.* 234

Why Porcelain Bridges Fail—The Remedy, *By George W. Schwartz, M.D., D.D.S.* 244

Ethics, - - - - *By Dr. F. W. Harnden.* 247

New Method of Forming an Inlay Matrix, *By Charles Channing Allen, D.D.S.* 251

A Useful Antiseptic - *By Bernard Bennette, L.D.S.* 253

Treatment of Bilateral Empyema of Maxillary Sinuses, *By George Zederbaum, D.D.S.* 254

Editorial, - - - - - - - - - - 257

Announcements, - - - - - - - - 258

Indents, - - - - - - - - - 264

